# Prevalence and incidence of type 1 diabetes in the world: a systematic review and meta-analysis

**DOI:** 10.34172/hpp.2020.18

**Published:** 2020-03-30

**Authors:** Majid Mobasseri, Masoud Shirmohammadi, Tarlan Amiri, Nafiseh Vahed, Hossein Hosseini Fard, Morteza Ghojazadeh

**Affiliations:** ^1^Endocrine Research Center, Tabriz University of Medical Sciences, Tabriz, Iran; ^2^Liver and Gastrointestinal Diseases Research Center, Tabriz University of Medical Sciences, Tabriz, Iran; ^3^Student Research Committee, Tabriz University of Medical Sciences, Tabriz, Iran; ^4^Emergency Medicine Research Team, Tabriz University of Medical Sciences, Tabriz, Iran; ^5^Research Center for Evidence-Based Medicine, Iranian EBM Centre: A Joanna Briggs Institute Affiliated Group, Tabriz University of Medical Sciences, Tabriz, Iran

**Keywords:** Diabetes mellitus, Incidence, Prevalence, Systematic review, Type 1, World

## Abstract

**Background:** Diabetes is referred to a group of diseases characterized by high glucose levels in blood. It is caused by a deficiency in the production or function of insulin or both, which can occur because of different reasons, resulting in protein and lipid metabolic disorders. The aim of this study was to systematically review the prevalence and incidence of type 1 diabetes in the world.

**Methods:** A systematic search of resources was conducted to investigate the prevalence and incidence of type 1 diabetes in the world. The databases of Medline (via PubMed and Ovid),ProQuest, Scopus, and Web of Science from January 1980 to September 2019 were searched to locate English articles. The located articles were screened in multiple levels of title, abstract,and full-text and final studies that met the inclusion criteria were retrieved and included in the study.

**Results:** From 1202 located articles, 193 studies were included in this systematic review. The results of meta-analysis showed that the incidence of type 1 diabetes was 15 per 100,000 people and the prevalence was 9.5% (95% CI: 0.07 to 0.12) in the world, which was statistically significant.

**Conclusion:** According to the results, the incidence and prevalence of type 1 diabetes are increasing in the world. As a result, insulin will be difficult to access and afford, especially in underdeveloped and developing countries.

## Introduction

Diabetes is referred to a group of diseases characterized by high glucose levels in blood. It is caused by a deficiency in the production or function of insulin or both, which can occur because of different reasons, resulting in protein and lipid metabolic disorders.^1^ The long-term effects of hypoglycemia are tissue and organ damage.^2^

Symptoms of diabetes include polyuria, thirst, vision disorders, and weight loss. In some cases there are more severe forms of diabetic ketoacidosis and hyperosmolar that may lead to stupor and coma. But most symptoms are not severe, which may cause damage or even failure of different organs in the long run and lead to irreparable injuries such as blindness, amputation, stroke and eventually death. Previously, type 1 diabetes was called insulin-dependent diabetes and it could happen at any age but is most common in children and young people.^3^

People with type 1 diabetes are not able to produce enough insulin. This type constitutes about 5%–10% of all cases of diabetes. In this type, the cellular destruction of beta cells occurs in the pancreas. In type 1 diabetes, the pancreas does not release any insulin. Since there is no epidemiologically accurate information on the prevalence and incidence of type 1 diabetes in the world and in the region, therefore, the present study was designed and implemented as a systematic review and meta-analysis, because of geopolitical map of the policy on the prevention and treatment of this disease can be done better.

## Materials and Methods

In this systematic review and meta-analysis, a systematic search of resources was conducted by a librarian (N.V.) to investigate the prevalence and incidence of type 1 diabetes (condition) in the people (population) of the world (context). The PICO of study based on the JBI protocol as CoCoPop for prevalence and incidence studies.

### Data sources and search strategy

The databases of Medline via (PubMed, Ovid), Embase, Scopus, Web of Science from January 1980 to September 2019 were searched to locate English articles. Also, SID, Magiran, and Barakat databases were searched for Persian studies. The grey literature and ongoing studies were searched using the following: OpenGrey, Google Scholar and for thesis and dissertations ProQuest and studies presented at conferences were also searched. Also, experts and professionals on this subject were reached and their opinions were gathered for information on published and unpublished studies. The search was performed using MESH and free keywords. The keywords selected for the search were: “type 1 diabetes”, “prevalence”, and “incidence” with this search strategy: (((“Diabetes Mellitus, Type 1”[Mesh]) OR ((((((((((((((((((((IDDM[Title/Abstract]) OR T1DM[Title/Abstract]) OR “Type 1 Diabetes”[Title/Abstract]) OR “Autoimmune Diabetes”[Title/Abstract]) OR “Juvenile Onset Diabetes”[Title/Abstract]) OR “Juvenile-Onset Diabetes”[Title/Abstract]) OR “Brittle Diabetes Mellitus”[Title/Abstract]) OR “brittle diabetes”[Title/Abstract]) OR “diabetes mellitus type 1”[Title/Abstract]) OR “diabetes mellitus type I”[Title/Abstract]) OR “diabetes type 1”[Title/Abstract]) OR “diabetes type I”[Title/Abstract]) OR “early onset diabetes mellitus”[Title/Abstract]) OR “insulin dependent diabetes”[Title/Abstract]) OR “juvenile diabetes”[Title/Abstract]) OR “juvenile diabetes mellitus”[Title/Abstract]) OR “type I diabetes”[Title/Abstract]) OR “type I diabetes mellitus”[Title/Abstract]) OR “Insulin Dependent Diabetes Mellitus”[Title/Abstract]) OR “Insulin-Dependent Diabetes Mellitus”[Title/Abstract]))) AND ((((“Prevalence”[Mesh]) OR ((Prevalence[Title/Abstract]) OR Prevalences[Title/Abstract]))) OR ((“Incidence”[Mesh]) OR ((Incidence[Title/Abstract]) OR Incidences[Title/Abstract]))). The complete search strategy of Medline and Embase is in Supplementary file 1.

### Inclusion and exclusion criteria

Inclusioncriteria for selecting studies include: 1. Articles published between 1980 and 2019; 2. Articles published in English and Persian. The exclusion criteria were: 1. Studies with no reported sample size; 2. Studies that had low quality; 3. Studies that were published before 1990.

### Study selection

The located articles were screened in multiple levels of title, abstract, and full-text and final studies that met the inclusion criteria were retrieved and included in the study. The studies were critically appraised by 2 subject specialists and low-quality studies were excluded. In cases of disagreements between two experts (M.M. and M.S.) at each stage of selection and appraisal, third person opinion was used.

### Quality appraisal

Articles were evaluated using the STROBE checklist. In this checklist, the minimum score was 2 and the maximum was 4. Finally, articles that received a score of 4 on checklist questions were included in the research, 128 articles earned 4 score, 46 articles earned 3 score and 19 articles earned 2 score and finally their data were extracted to perform the meta-analysis.

### Data extraction and quality assessment

The information extracted from the articles were entered in the extraction form. Extracted data included: first author, year of publication, country of study, sample size, and incidence of diabetes in the studies.

### Statistical analysis

Statistical analysis was performed using CMA v.2.0 software and *P* value less than 0.05 was considered as significant. The binomial distribution was used to calculate the variance. Weighted mean was used to combine the prevalence rate of different studies. Meta-analysis was used to obtain the incidence of type 1 diabetes. The heterogeneity between studies was assessed by Cochran (Q) and I^2^ statistics, which expressed the percentage of variation between studies. Random effects model was used to calculate the overall and pooled effect size.

## Results

### Search results and study characteristics

**Figure 1:**
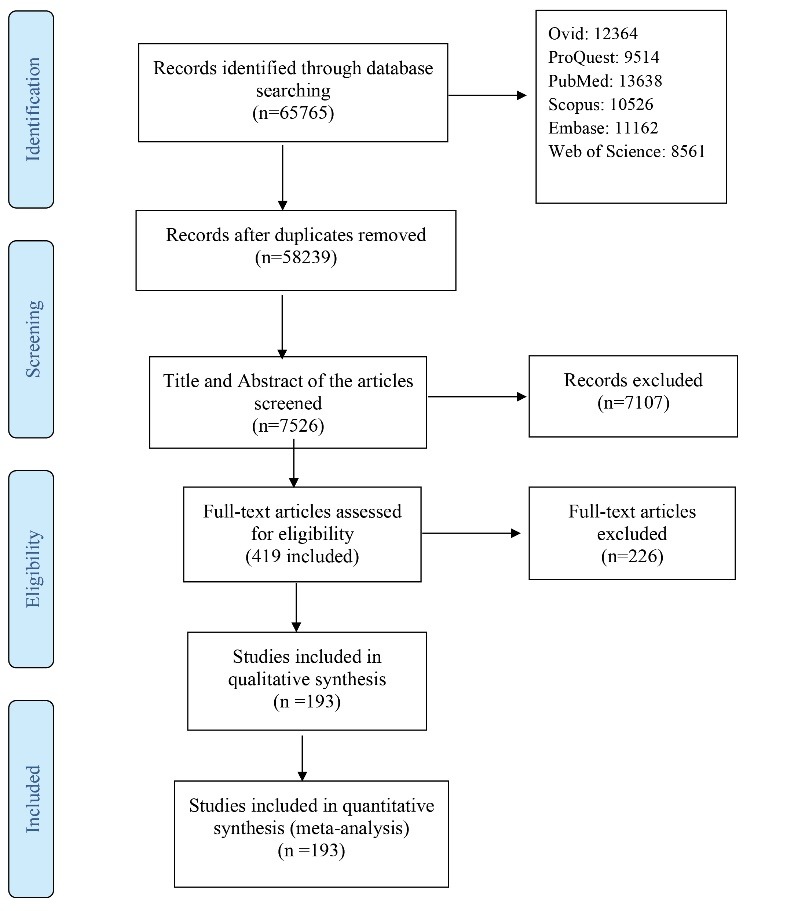
Flow chart of systematic review.

In a systematic search of sources, 65 765 articles were identified. A total of 58 239 articles were duplicates, and 7107 were excluded after reviewing the title and abstract of the articles. After reviewing the full-text articles, 49 articles were excluded. Finally, 193 studies were included in the systematic review and meta-analysis. Figure 1 shows the identified and retrieved articles in the study. Tables 1, 2 and 3 show the specifications of the articles that were studied.

**Table 1:** Characteristics of studies incidence of type 1 diabetes

**Study**	**Year**	**Country**	**Continent**	**Sample size**	**Incidence per 10000**
Abduljabbar et al^4^	2010	Saudi Arabia	Asia	1028	0.02752
Abdul-Rasoul et al^5^	2002	Kuwait	Asia	760	0.02018
Abellana et al^6^	2009	Spain	Europe	448	0.0118
Ajlouni et al^7^	1999	Jordan	Asia	123	0.0032
				107	0.0028
				138	0.0036
Alaghehbandan et al^8^	2006	Canada	USA	716	0.019
Alemu et al^9^	2009	Ethiopia	Africa	81	0.0021
Algert CS et al^10^	2009	Australia	Asia	605	0.016
Altobelli et al^11^	1998	Italy	Europe	355	0.00934
Arpi et al^12^	2002	Catania	Europe	470	0.01238
Aschner et al^13^	2014	America	USA	279	0.00731
Bahíllo et al^14^	2007	Spain	Europe	835	0.02222
Barat et al^15^	2008	French	Europe	510	0.01347
Battelino and Kržišnik^16^	1998	Slovenia	Europe	305	0.008
Berhan et al^17^	2011	Sweden	Europe	1612	0.0439
Bessaoud et al^18^	1990	Algeria	Africa	168	0.0044
Bizzarri et al^19^	2010	Italy	Europe	593	0.01568
Blanchard et al^20^	1997	Canada	USA	768	0.0204
Blumenfeld et al^21^	2014	Asia	Asia	433	0.0114
Bratina et al^22^	2001	Bosnia and Herzegovina	Europe	325	0.00854
Bruno et al^23^	1990	Italy	Europe	259	0.00678
Bruno et al^24^	1993	Italy	Europe	256	0.0067
Bruno et al^25^	1997	Italy	Europe	282	0.00739
Bruno et al^26^	2001	Italy	Europe	297	0.00778
Bruno et al^27^	2009	Italy	Europe	354	0.0093
Bruno et al^28^	2010	Italy	Europe	465	0.01226
Bruno et al^29^	2013	Italy	Europe	1644	0.0448
Calle-Pascual et al^30^	1993	Spain	Europe	565	0.01493
Calori et al^31^	1990	Italy	Europe	253	0.00663
Campbell-Stokes and Taylor^32^	2005	New Zealand	Europe	675	0.0179
Cardwell et al^33^	2006	Ireland	Europe	925	0.0247
Carrasco et al^34^	1996	Chile	USA	90	0.00236
Carrasco et al^35^	2006	Chile	USA	251	0.00658
Carrasco et al^36^	2006	Chile	USA	154	0.00402
Casu et al^37^	2004	Sardinia	Europe	1433	0.0388
Cherubini et al^38^	1994	Italy	Europe	309	0.0081
Chong et al^39^	2007	Australia	Asia	731	0.0194
Cinek et al^40^	2000	Czech Republic	Europe	384	0.0101
Cinek et al^41^	2003	Czech Republic	Europe	444	0.0117
Compés et al^42^	2013	Spain	Europe	723	0.0192
Cotellessa et al^43^	2003	Italy	Europe	476	0.01256
Crow et al^44^	1991	England	Europe	560	0.0148
				508	0.0134
Dabelea et al^45^	2009	Navajo nation	USA	86	0.00224
				841	0.0224
				1452	0.03934
Dacou-Voutetakis et al^46^	1995	Greece	Europe	239	0.00625
Demirbilek et al^47^	2013	Turkey	Asia	275	0.0072
Derraik et al^48^	2012	New Zealand	Europe	845	0.0225
Dziatkowiak et al^49^	2002	Poland	Europe	316	0.0083
				244	0.0064
				301	0.0079
Ehehalt et al^50^	2012	Europe	Europe	579	0.0153
Ehehalt et al^51^	2009	Italy	Europe	560	0.0148
Ehehalt et al^52^	2012	Europe	Europe	579	0.0153
El-Ziny et al^53^	2014	Egypt	Africa	119	0.0031
Feltbower et al^54^	2002	UK	Europe	493	0.013
Ferreira et al^55^	1993	Brazil	USA	290	0.0076
Forga et al^56^	2013	Spain	Europe	331	0.0087
Formosa et al^57^	2012	Malta	Africa	821	0.02186
Frazer De Llado et al^58^	1998	Puerto Rico	USA	679	0.018
Frongia et al^59^	1997	Italy	Europe	1411	0.0382
Gardner et al^60^	1997	USA	USA	701	0.0186
Charkaluk et al^61^	2002	France	Europe	364	0.00958
Giralt et al^62^	2001	Spain	Europe	973	0.026
Goday et al^63^	1992	Spain	Europe	407	0.0107
Gong et al^64^	2013	China	Asia	56	0.00145
Gopinath et al^65^	2008	Sweden	Europe	914	0.02438
Gorham et al^66^	1993	USA	USA	801	0.0213
Grabauskas et al^67^	1991	Lithuania	Europe	256	0.0067
Green and Patterson^68^	2001	Hungary	Europe	686	0.0182
Harjutsalo et al^69^	2008	Finland	Europe	1577	0.0429
Harjutsalo et al^70^	2013	Finland	Europe	2264	0.0629
Huen et al^71^	2000	Hong Kong	Asia	54	0.0014
Jarosz-Chobot et al^72^	2010	Poland	Europe	375	0.00987
Jarosz-Chobot et al^73^	2011	Poland	Europe	388	0.0102
Ji et al^74^	2010	Sweden	Europe	27	0.00071
Kadiki and Moawad^75^	1994	Libya	Africa	335	0.0088
Kadiki et al^76^	1996	Libya	Africa	343	0.009
Karvonen et al^77^	1996	Finland	Europe	1319	0.0356
Karvonen et al^78^	2000	China & Venezuela	Asia	4	0.0001
Karvonen et al^79^	1997	Finland	Europe	1507	0.0409
Kida et al^80^	1999	Japan	Asia	58	0.0015
Koton^81^	2007	Asia	Asia	305	0.008
Kulaylat and Narchi^82^	2000	Saudi Arabia	Asia	437	0.0115
Lammi et al^83^	2007	Finland	Europe	601	0.0159
Larenas et al^84^	1996	Chile	USA	49	0.00127
Lawrence et al^85^	2014	USA	USA	914	0.0244
Legault and Polychronakos^86^	2006	Canada	USA	568	0.015
Libman et al^87^	1998	USA	USA	631	0.0167
Lin et al^88^	2014	Taiwan	Asia	128	0.00334
Lipman^89^	1993	USA	USA	494	0.01302
Lipman et al^90^	2002	USA	USA	504	0.0133
Lipman et al^91^	2006	USA	USA	560	0.0148
Lipman et al^92^	2013	USA	USA	642	0.017
Lipton et al^93^	2002	USA	USA	575	0.0152
Lisbôa et al^94^	1998	Brazil	USA	455	0.012
Li et al^95^	2000	China	Asia	22	0.00056
Lora-Gómez et al^96^	2005	Spain	Europe	635	0.0168
Mamoulakis et al^97^	2003	Crete	Europe	233	0.0061
Martinucci et al^98^	2002	Belarus	Europe	176	0.0046
Mauny et al^99^	2005	France	Europe	230	0.00603
Mayer-Davis et al^100^	2009	USA	USA	594	0.0157
Mazzella et al^101^	1994	Italy	Europe	445	0.01172
Metcalfe and Baum^102^	1991	Britain	Europe	512	0.0135
Michalková et al^103^	2004	Slovakia	Europe	529	0.01396
Morales-Pérez et al^104^	2000	Spain	Europe	485	0.0128
Muiña et al^105^	2012	Spain	Europe	1031	0.0276
Muntoni et al^106^	1992	Sardinia	Europe	911	0.0243
Muntoni et al^107^	1997	Italy	Europe	1255	0.0338
Neu et al^108^	1997	German	Europe	440	0.0116
Neu et al^109^	2001	Europe	Europe	474	0.0125
Newhook et al^110^	2004	Canada	USA	1331	0.03593
Newhook et al^111^	2008	Canada	USA	1300	0.03508
Newhook et al^112^	2012	Canada	USA	1394	0.0377
Ostrauskas et al^113^	2011	Lithuania	Europe	316	0.0083
Patterson et al^114^	2000	Macedonia	Europe	123	0.0032
Patterson et al^115^	2001	Finland	Europe	1482	0.0402
Peter^116^	2007	Bahamas	USA	384	0.0101
Pinelli et al^117^	1998	Italy	Europe	407	0.0107
Pishdad^118^	2005	Iran	Asia	120	0.00314
Podar et al^119^	1992	Estonia	Europe	448	0.0118
Polanska et al^120^	2014	Poland	Europe	452	0.01192
Prisco et al^121^	1996	Italy	Europe	232	0.00607
Pronina et al^122^	2008	Moscow	Europe	489	0.0129
Pundziute-Lyckå et al^123^	2003	Lithuania	Europe	361	0.0095
				263	0.0069
Radosevic et al^124^	2013	Bosnia and Herzegovina	Europe	286	0.0075
		Slovenia		474	0.0125
Ramachandran et al^125^	1996	India	Asia	399	0.0105
Rami et al^126^	2001	Austria	Asia	342	0.00899
Rangasami et al^127^	1997	Scotland	Europe	896	0.0239
Serrano Río et al^128^	1990	Spain	Europe	429	0.0113
Roche et al^129^	2002	Ireland	Europe	627	0.0166
Rosenbauer et al^130^	1999	Europe	Europe	309	0.0081
Aude Rueda et al^131^	1998	Mexico	USA	44	0.00115
Rytkönen et al^132^	2003	Finland	Europe	1383	0.0374
Samardzic et al^133^	2010	Montenegro	Europe	508	0.0134
Samuelsson et al^134^	1994	Sweden	Europe	944	0.0252
Santos et al^135^	2001	Chile	USA	157	0.00411
Sasaki and Okamoto^136^	1992	Japan	Asia	64	0.00168
				77	0.002
Schober et al^137^	1995	Australia	Asia	301	0.0079
Schober et al^138^	2009	Austria	Asia	694	0.0184
Schoenle et al^139^	2001	Switzerland	Europe	399	0.0105
Scott et al^140^	1992	New Zealand	Europe	482	0.0127
Sebastiani et al^141^	1996	Italy	Europe	301	0.0079
Sella et al^142^	2010	Asia	Asia	481	0.01269
Sereday et al^143^	1994	Argentina	USA	2694	0.0759
Shaltout et al^144^	2002	Kuwait	Asia	757	0.0201
Shamis et al^145^	1997	Asia	Asia	278	0.0073
López Siguero et al^146^	1997	Malaga	Europe	541	0.0143
Sipetic et al^147^	2013	Serbia	Europe	395	0.0104
Skordis and Hadjiloizou^148^	1997	Greece	Europe	399	0.0105
Skordis et al^149^	2002	Greece	Europe	430	0.01132
Skordis et al^150^	2012	Cyprus	Asia	473	0.01246
Skrivarhaug et al^151^	2014	Norway	Europe	1215	0.0327
Smith et al^152^	2007	USA	USA	683	0.0181
Staines et al^153^	1993	UK	Europe	519	0.0137
Staines et al^154^	1997	Pakistan	Asia	39	0.00102
Stipancic et al^155^	2008	Croatia	Europe	338	0.00887
Svensson et al^156^	2002	Denmark	Europe	731	0.0194
Svensson et al^157^	2008	Denmark	Europe	827	0.022
Swai et al^158^	1993	Tanzania	Africa	58	0.0015
Tahirovic et al^159^	2007	Bosnia and Herzegovina	Europe	271	0.0071
Taplin et al^160^	2005	New South Wales	Asia	786	0.0209
Teeäär et al^161^	2009	Estonia	Europe	649	0.0172
Thunander et al^162^	2008	Sweden	Europe	1397	0.0378
Torffvit et al^163^	2007	Sweden	Europe	482	0.0127
Toth et al^164^	1997	Canada	USA	962	0.0257
Toumba et al^165^	2007	Cyprus	Asia	452	0.0119
Tran et al^166^	2014	Australia	Asia	827	0.022
Tuchinda et al^167^	2002	Thailand	Asia	63	0.00165
Tull et al^168^	1991	Virgin Islands	USA	286	0.0075
Tuomilehto et al^169^	1991	Finland	Europe	1219	0.0328
Tuomilehto-Wolf et al^170^	1991	Estonia	Europe	407	0.0107
Tuomilehto et al^171^	1992	Finland	Europe	1305	0.0352
Tuomilehto et al^172^	1992	Finland	Europe	1031	0.0276
Tuomilehto et al^173^	1993	Mauritius	Africa	81	0.0021
Tuomilehto et al^174^	199	Finland	Europe	1369	0.037
Tzaneva et al^175^	1998	Bulgaria	Europe	241	0.00632
Vandewalle et al^176^	1997	Belgium	Europe	448	0.0118
Vehik^177^	2007	Colorado	USA	560	0.0148
Verge et al^178^	1994	Australia	Asia	549	0.0145
Vichi et al^179^	2014	Italy	Europe	508	0.0134
Vlajinac et al^180^	1995	Serbia	Europe	294	0.0077
Vos et al^181^	1996	Netherland	Europe	753	0.02
Wadsworth et al^182^	1995	England	Europe	354	0.0093
Washington et al^183^	2012	USAVirgin Islands	USA	579	0.0153
Willis et al^184^	2002	New Zealand	Europe	757	0.02012
Wong^185^	1994	China	Asia	65	0.0017
Wong et al^186^	1993	Hong Kong	Asia	77	0.002
Yang et al^187^	1998	China	Asia	18	0.00048
Yang et al^188^	2005	China	Asia	18	0.00047
Zalutskaya et al^189^	2004	Gomel area	Europe	300	0.00786
		Minsk area		127	0.00332
Zhao et al^190^	1999	England	Europe	564	0.0149
Zhao et al^191^	2014	China	China	119	0.0031
Zubkiewicz-Kucharska and Noczyńska^192^	2010	Poland	Europe	471	0.01241

**Table 2:** Prevalence and incidence of type 1 diabetes in the world

	**Prevalence Per 10 000**	**Incidence Per 100 000**
World	5.9	15
Asia	9.6	15
Africa	5.3	8
Europe	2.12	
America	3.9	20

**Table 3:** Characteristics of studies prevalence of type 1 diabetes

**Study**	**Country**	**Sample Size**	**Prevalence Per 100 000**
Akazawa^193^	Japan	40	10
Akesen et al^194^	Turkey	26	67
Al-Herbish et al^195^	Saudi Arabia	42	109.5
Aschner et al^13^	America	2827	8000
Bessaoud et al^18^	Algeria	10	27
Dabelea et al^45^	Navajo nation	40	11
		31	81
		106	278
Dabelea et al^196^	USA	57	148
Ehehalt et al^51^	Italy	3761	11000
Elamin et al^197^	Sudan	17	42.98
El-Ziny et al^53^	Egypt	10	26.8
Eriksson et al^198^	Finland	1009	2700
Evans et al^199^	Scotland	6592	22000
Frongia et al^59^	Italy	176	459
Garancini et al^200^	Italy	31	80
Gujral et al^201^	UK	29	75
Jorge et al^202^	Portugal	49	128
Kemper et al^203^	USA	70	183
Mayer-Davis et al^100^	USA	218	570
Moussa et al^204^	Kuwait	103	269.9
Ostrauskas^205^	Lithuania	31	80.64
Ostrauskas and Žalinkevičius^206^	Lithuania	27	70.23
Peter et al^116^	Bahamas	12	31
Pettitt et al^207^	USA	74	193
Ramachandran et al^208^	India	10	26
Rangasami et al^127^	Scotland	58	150
Scott et al^140^	New Zealand	44	115
López Siguero et al^146^	Malaga	297	780
Soliman et al^209^	Oman	50	13.25
Songini et al^210^	Sardinia	46	119
Wong^185^	China	30	8.3
Wu et al^211^	New Zealand	87	227

### Prevalence and incidence of type 1 diabetes in Asia

**Figure 2:**
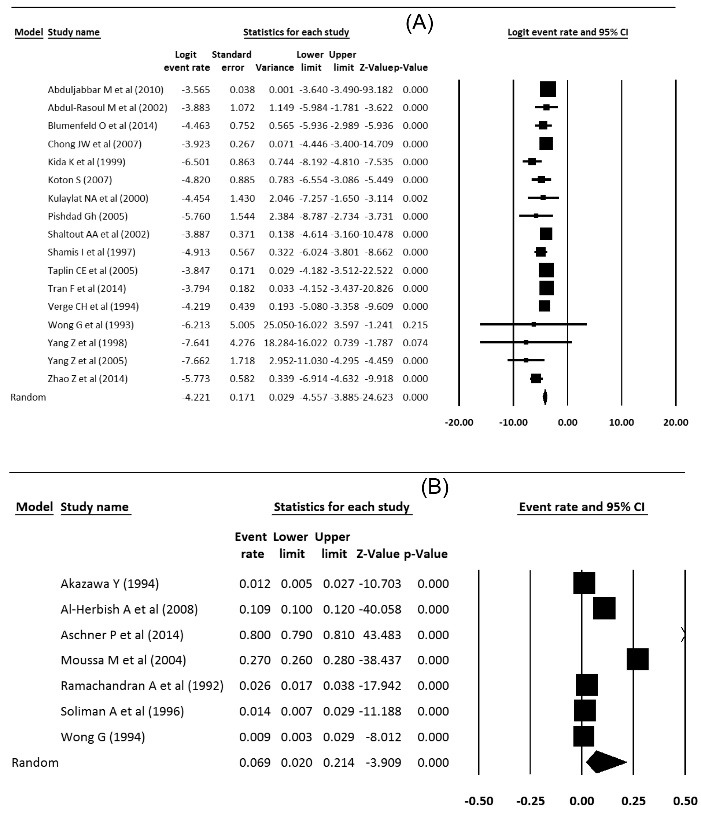
(A) Incidence and (B) prevalence of type 1 diabetes in Asia.

Prevalence and incidence of type 1 diabetes were extracted from meta-analysis studies. In type 1 diabetes incidence, the heterogeneity between studies in the meta-analysis was significant (Q = 50.51; df = 16; *P* < 0.001; I^2^ = 68.33), but in the prevalence of diabetes 1, the heterogeneity was not significant (Q = 5220; *df* = 6; *P* < 0.001; I^2^ = 99.88). The incidence of type 1 diabetes in Asia was 15 per 100 000 population, which was statistically significant (Incidence = 0.015, 95% CI = 0.010 to 0.021, *P* < 0.001), and the prevalence of type 1 diabetes was 6.9 per 10 000 people, which was statistically significant (Prevalence = 0.069, 95% CI = 0.020 to 0.214, *P* < 0.001). Figures 2A and 2B show the forest plot of prevalence and incidence of type 1 diabetes in Asia.

### Prevalence and incidence of type 1 diabetes in Africa

**Figure 3:**
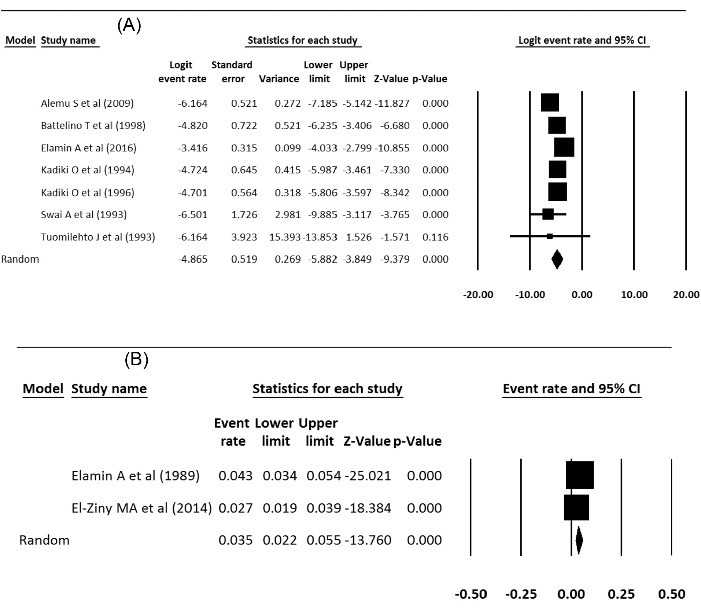
(A) Incidence and (B) prevalence of type 1 diabetes in Africa.

Prevalence and incidence of type 1 diabetes were extracted from meta-analysis studies. In type 1 diabetes incidence, the heterogeneity between studies in the meta-analysis was not significant (Q = 23.79; *df* = 6; *P* < 0.001; I^2^ = 74.78) and in the prevalence of diabetes 1, the heterogeneity was not significant too, (Q = 4.4; *df* = 1; *P* < 0.001; I^2^ = 77.27). The incidence of type 1 diabetes in Africa was 8 per 100 000 population, which was statistically significant (Incidence = 0.008, 95% CI = 0.003 to 0.021 *P* < 0.001), and the prevalence of type 1 diabetes was 3.5 per 10 000 people, which was not statistically significant (prevalence = 0.035, 95% CI: 0.022 to 0.055, *P* < 0.001). Figures 3A and 3B show the forest plot of prevalence and incidence of type 1 diabetes in Africa.

### Prevalence and incidence of type 1 diabetes in Europe

**Figure 4:**
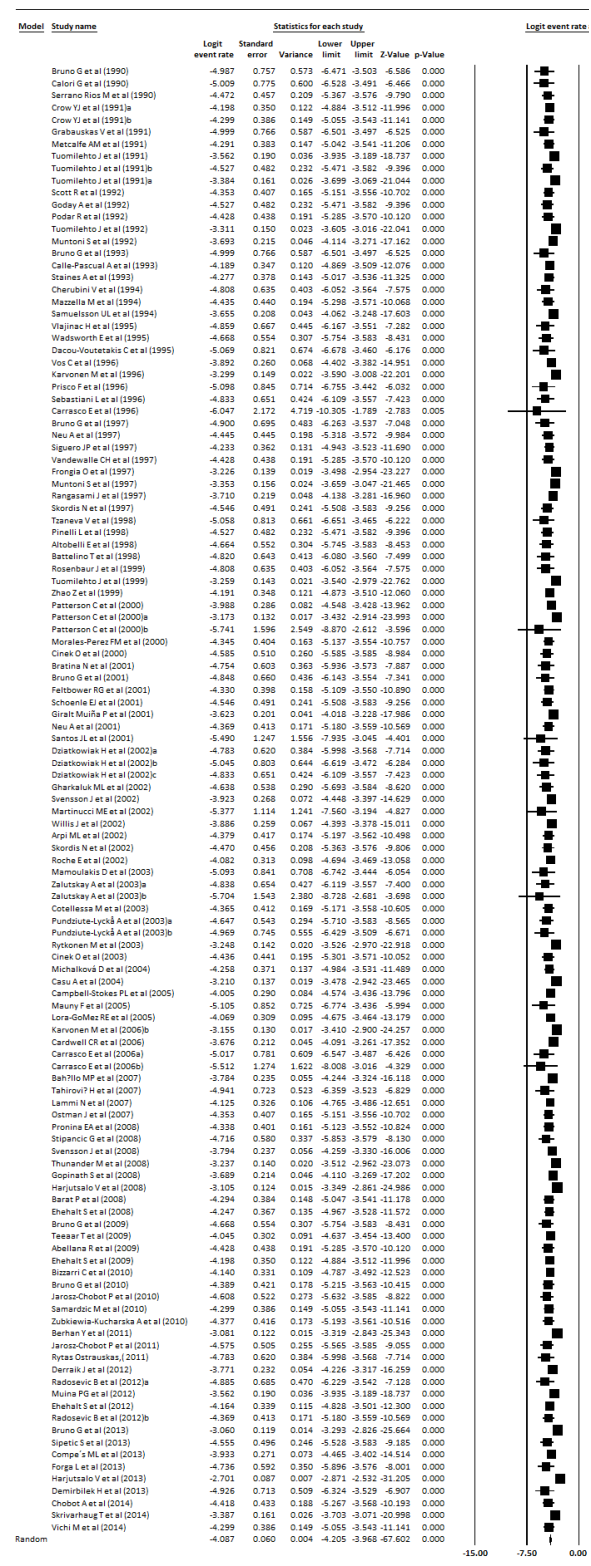
Incidence of type 1 diabetes in Europe.

**Figure 5:**
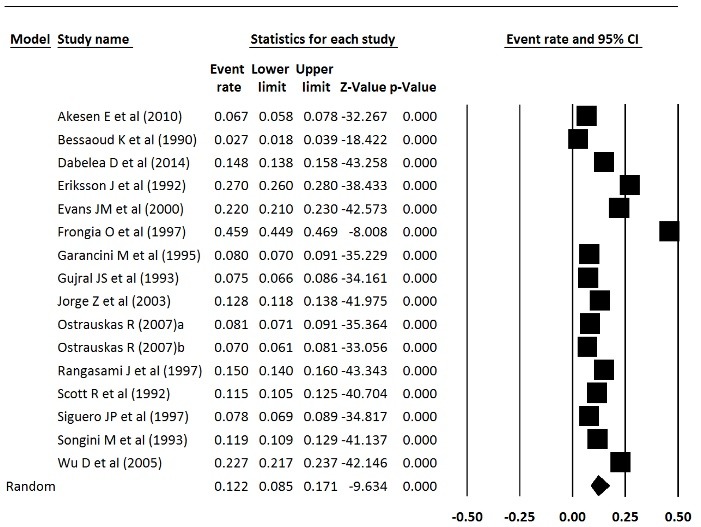
Prevalence of type 1 diabetes in Europe.

Prevalence and incidence of type 1 diabetes were extracted from meta-analysis studies. In type 1 diabetes incidence, the heterogeneity between studies in the meta-analysis was significant (Q = 895.56, *df* = 96, *P* < 0.001, I^2^ = 89.28) but in the prevalence of diabetes 1, the heterogeneity was not significant, (Q = 5792.85, *df* = 15, *P* < 0.001, I^2^ = 99.74). The incidence of type 1 diabetes in Europe was 15 per 100 000 population, which was statistically significant (Incidence = 0.015, 95% CI = 0.013 to 0.018, *P* < 0.001), and the prevalence of type 1 diabetes was 12.2 per 10 000 people, which was statistically significant (Prevalence = 0.122, 95% CI = 0.085 to 0.171, *P* < 0.001). Figures 4 and 5 show the forest plot of prevalence and incidence of type 1 diabetes in Europe.

### Prevalence and incidence of type 1 diabetes in America

**Figure 6:**
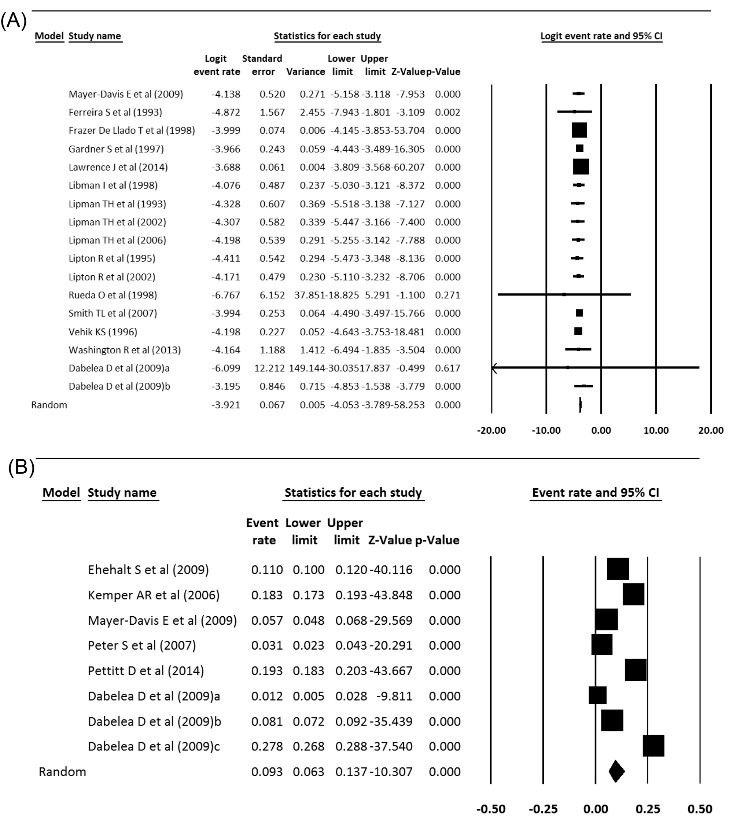
(A) Incidence and (B) prevalence of type 1 diabetes in America.

Prevalence and incidence of type 1 diabetes were extracted from meta-analysis studies. In type 1 diabetes incidence, the heterogeneity between studies in the meta-analysis was significant (Q = 18.88, *df* = 16, *P* = 0.27, I^2^ = 15.28) and in the prevalence of diabetes 1, the heterogeneity was significant too, (Q = 1120.79, df = 7, *P* < 0.001, I^2^ = 99.38). The incidence of type 1 diabetes in America was 20 per 100 000 population, which was statistically significant (Incidence = 0.020, 95% CI = 0.010 to 0.021, *P* < 0.001), and the prevalence of type 1 diabetes was 12.2 per 10 000 people, which was statistically significant (Prevalence = 0.093, 95% CI = 0.063 to 0.137, *P* < 0.001). Figures 6A and 6B show the forest plot of prevalence and incidence of type 1 diabetes in America. A sensitivity analysis was done for Incidence of type 1 diabetes in America based on excluding studies with too wide CIs. Sensitivity analysis’s results show that the incidence of type 1 diabetes in America is 19 per 100 000 population, which is statistically significant (Incidence = 0.019, 95% CI = 0.016 to 0.022, *P* < 0.001).

### Prevalence and incidence of type 1 diabetes in the world

**Figure 7:**
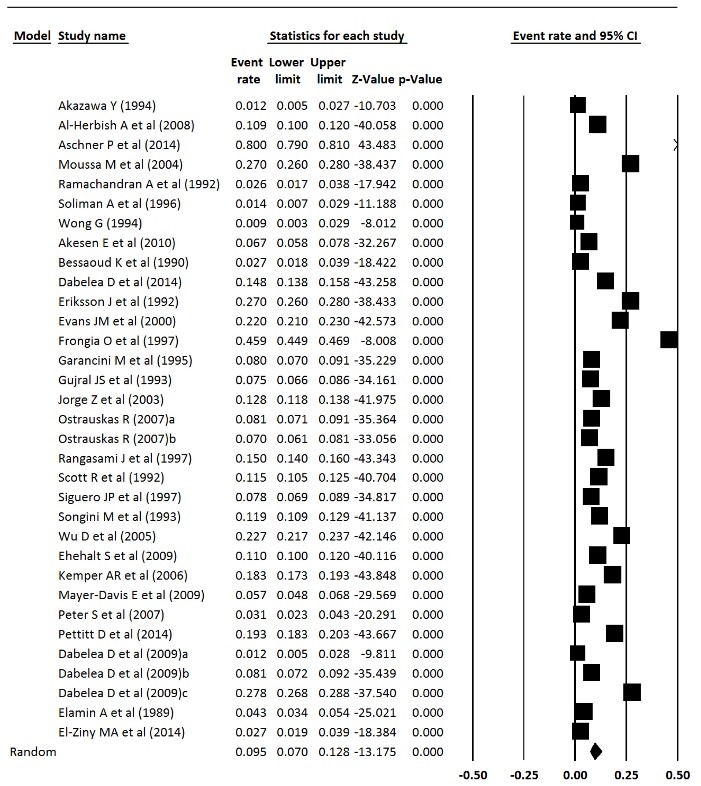
Prevalence of type 1 diabetes in the world.

Prevalence and incidence of type 1 diabetes were extracted from meta-analysis studies. In type 1 diabetes incidence, the heterogeneity between studies in the meta-analysis was significant (Q = 1020.30, df = 137, *P* < 0.001, I^2^ = 86.57) and in the prevalence of diabetes 1, the heterogeneity was significant too, (Q = 14760.32, df = 32, *P* < 0.001, I^2^ = 99.78). The incidence of type 1 diabetes in world was 15 per 100 000 population, which was statistically significant (Incidence = 0.015, 95% CI = 0.013 to 0.017, *P* < 0.001), and the prevalence of type 1 diabetes was 9.5 per 10 000 people, which was statistically significant (prevalence = 0.095, 95% CI = 0.070 to 0.128, *P* < 0.001). Figure 7 shows the forest plot of prevalence and incidence of type 1 diabetes in the world.

### Publication bias

In order to assess the publication bias, Eggers Regression test was used. Based on the results, the population bias between studies was not significant (t-value = 1.26, *df* 93, *P* = 0.21).

### Meta-Regression

**Figure 8:**
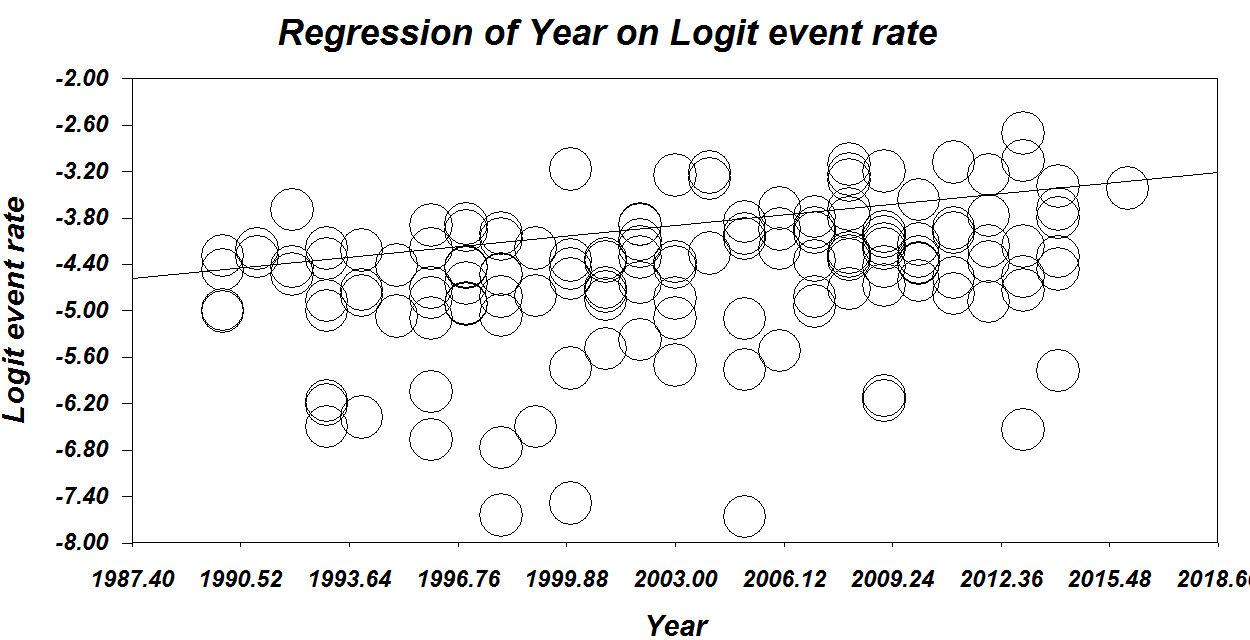
The meta-regression plot.

Meta-regression was used to determine the effect of time on type 1 diabetes incidence. The results showed that the incidence of type 1 diabetes has increased over time. The meta-regression plot is shown in Figure 8.

## Discussion

The global trend of increasing prevalence of type 1 diabetes, with multiple etiologies, operates through multiple mechanisms. In the present study, data were extracted from 193 articles between 1990 and 2019. The results showed that the incidence of type 1 diabetes in continental subgroups (Asia, Africa, Europe, and America) was 15 per 100 000, 8 per 100 000, 15 per 100 000 and 20 per 100, respectively. Also, the global prevalence of continental subtypes of type 1 diabetes in the above regions was, 6.9 per 10 000, 3.5 per 10 000, and 12.2 per 10 000, respectively.

Relative differences between obtained results and previous statistics may be due to different research time periods and new global population status. Especially in recent years (social, political and economic migration), the changing global climate coupled with new policies and sanctions that have led to poorer middle-income and low-income countries.^212^

The pathogenesis of type 2 diabetes is known, which is associated with different genes and the involvement of multiple factors. Type 2 diabetes can be prevented and treated by removing or reducing these factors. Most of the warnings of national and international health bodies and diabetes associations are based on lifestyle changes and stress reduction that can prevent diabetes.^213^

But in type 1 diabetes, that make up 5 to 15 percent of diabetics and often involve children, Prevention ways have not yet been defined. However, screening of type 1 diabetes in prone families in relation to autoantibodies has recently been proposed. Also, clinical studies on the prevention of type 1 diabetes have been conducted.^214^

If one foot was amputated every 30 seconds, today it’s every 15 seconds. Need for dialysis equipment will increase. The CCU and ICU beds will be full of stroke and myocardial infarction patients. The population of the blind increases and unfortunately, new, effective, and less complicated treatments become more expensive.^215^

The disease shows a significant increase in glucose and possibly DKA. These patients definitely need insulin due to the pathogenesis of insulin deficiency. Manufacturing and production of insulin (traditional insulins and analog insulins) and insulin pumps, despite being inexpensive in producing countries, is shipped to low- and middle-income countries for high prices which is a major problem for the managing of type 1 diabetes patients. Certainly, uncontrolled hyperglycemia in type 1 diabetic patients will make all the problems more severe.^216^

### Limitations

One of the limitations of the study was the poor quality of some articles and, despite a careful search, the lack of access to some of the full text of the published articles.

## Conclusion

According to the results, the incidence and prevalence of type 1 diabetes are increasing in the world. As a result, insulin will be difficult to access and afford, especially in underdeveloped and developing countries. Thus, warnings about this can help international organizations and countries to plan for preventive measures.

## Ethical approval

This research was approved by the Local Ethics Committee with No. 61701.

## Competing interests

The authors declare that they have no competing interests.

## Funding

This article was supported by the Research Center for Evidence-Based Medicine, and the Research Vice-Chancellor of Tabriz University of Medical Sciences.

## Authors’ contributions

Concept: MM. Study design: MSH and TA. Systematic search: NV. Critical reviews: MM and TA. Data extraction: MSH and MGH. Data analysis: MGH and HHF. Writing: NV, TA and MM. All authors had primary responsibility for the final content of the manuscript and read and approved the final manuscript.

## Acknowledgments

Special thanks to the Research Vice-Chancellor of Tabriz University of Medical Sciences for financial support for this study.

## Supplementary Materials

Supplementary file 1 contains search strategy.
