# Corrigendum to: Prevalence and incidence of type 1 diabetes in the world: a systematic review and meta-analysis Mobasseri M, Shirmohammadi M, Amiri T, Vahed N, Hosseini Fard H, Ghojazadeh M. Health Promot Perspect. 2020 Mar 30;10(2):98-115. doi: 10.34172/hpp.2020.18

**DOI:** 10.34172/hpp.43143

**Published:** 2024-07-29

**Authors:** Majid Mobasseri, Masoud Shirmohammadi, Tarlan Amiri, Nafiseh Vahed, Hossein Hosseini Fard, Morteza Ghojazadeh

**Affiliations:** ^1^Endocrine Research Center, Tabriz University of Medical Sciences, Tabriz, Iran; ^2^Liver and Gastrointestinal Diseases Research Center, Tabriz University of Medical Sciences, Tabriz, Iran; ^3^Student Research Committee, Tabriz University of Medical Sciences, Tabriz, Iran; ^4^Emergency Medicine Research Team, Tabriz University of Medical Sciences, Tabriz, Iran; ^5^Research Center for Evidence-Based Medicine, Iranian EBM Centre: A Joanna Briggs Institute Affiliated Group, Tabriz University of Medical Sciences, Tabriz, Iran

## Corrigendum

 Based on the comments we received from the readers of our article entitled “Prevalence and incidence of type 1 diabetes in the world: a systematic review and meta-analysis”, published in *Health Promotion Perspectives*, we rechecked the whole of the article and its associated data set, and identified a set of errors and missing attributions that should be corrected as follows:

 In the results section, heterogeneity between studies for the prevalence of type 1 diabetes in Asia, Africa, and Europe was incorrectly written as non-significant, while it should have been reported as significant. Also, heterogeneity between the studies in Africa was written as non-significant in the main text, which should have been reported as statistically significant.

 In terms of the incidence of type 1 diabetes in America, there is a non-significant heterogeneity, which was incorrectly written as significant in the text. Additionally, in the published paper, there are some incorrect values in Table 3; however, the values used for the meta-analysis are correct. In this table, the correct prevalence values per 100 000 reported from the studies of Mayer-Davis et al,^1^ Ehehalt et al,^2^ Erikson et al,^3^ Evans et al,^4^ and Lopez Siguero et al^5^ are 57, 110, 270, 220, and 78, respectively.

 Furthermore, Moussa et al^6,7^ conducted two studies to investigate the prevalence of type 1 and type 2 diabetes among Kuwaiti children, and in our article, instead of the reference of the study on type 1 diabetes,^6^ the reference of the study considering type 2 diabetes was cited.^7^ In addition, Peter had two studies, within which the trend of type 1 diabetes,^8^ and the prevalence and incidence of type 1 diabetes in the Bahamas^9^ were determined. In our meta-analysis, the first study was mistakenly cited instead of the second study.

 In the studies conducted by Dabelea et al,^10^ Kemper et al,^11^ Ashner et al,^12^ and Garancini et al,^13^the prevalence estimates reported were for overall diabetes, type 1 diabetes, or type 2 diabetes. In our study, however, those estimates were incorrectly extracted as the estimates for overall diabetes or type 2 diabetes. So, the corrections were as follow: for the study of Kemper et al,^11^ the errors were corrected and the type 1 diabetes data were extracted; the study of Dabelea et al^10^ was replaced with another publication with more complete information^14^; for the study of Garancini et al,^13^ the data on type 1 diabetes were unclear and insufficient to be included in our analysis, and was therefore excluded from the meta-analysis; in the study of Ashner et al,^12^ the prevalence estimate of type 1 diabetes was not reported, and was thus excluded from our analysis.

**Table 3:** Characteristics of studies prevalence of type 1 diabetes

**Study**	**Country**	**Sample Size**	**Prevalence Per 100000**
Akazawa^193^	Japan	40	10
Akesen et al^194^	Turkey	26	67
Al-Herbish et al^195^	Saudi Arabia	42	109.5
Aschner et al^13^	America	2827	8000
Bessaoud et al^18^	Algeria	10	27
Dabelea et al^45^	Navajo nation	40	11
		31	81
		106	278
Dabelea et al^196^	USA	57	148
Ehehalt et al^51^	Italy	3761	110
Elamin et al^197^	Sudan	17	42.98
El-Ziny et al^53^	Egypt	10	26.8
Eriksson et al^198^	Finland	1009	270
Evans et al^199^	Scotland	6592	220
Frongia et al^59^	Italy	176	459
Garancini et al^200^	Italy	31	80
Gujral et al^201^	UK	29	75
Jorge et al^202^	Portugal	49	128
Kemper et al^203^	USA	70	183
Mayer-Davis et al^100^	USA	218	57
Moussa et al^204^	Kuwait	103	269.9
Ostrauskas^205^	Lithuania	31	80.64
Ostrauskas and Žalinkevičius^206^	Lithuania	27	70.23
Peter et al^116^	Bahamas	12	31
Pettitt et al^207^	USA	74	193
Ramachandran et al^208^	India	10	26
Rangasami et al^>127^	Scotland	58	150
Scott et al^140^	New Zealand	44	115
López Siguero et al^146^	Malaga	297	78
Soliman et al^209^	Oman	50	13.25
Songini et al^210^	Sardinia	46	119
Wong^185^	China	30	8.3
Wu et al^211^	New Zealand	87	227

 In addition, the estimated prevalence of type 1 diabetes reported by Elamin et al^15^ in Sudan, was extracted incorrectly. We thus corrected the errors and repeated the meta-analyses, and found that the estimated prevalence of type 1 diabetes was 0.038 (95% CI: 0.017 to 0.084, *P *< 0.001) in Asia (Figure 1 [the corrected form of Figure 2-B in the original article]), 0.052 (95% CI: 0.015 to 0.168, *P *< 0.001) in Africa (Figure 2 [the corrected form of Figure 5-B in the original article]), 0.125 (95% CI: 0.086 to 0.177, *P *< 0.001) in Europe (Figure 3 [the corrected form of Figure 3-B in the original article]), 0.050 (95% CI: 0.036 to 0.070, *P *< 0.001) in America (Figure 4 [the corrected form of Figure 6-B in the original article]), and 0.075 (95% CI: 0.051 to 0.110, *P *< 0.001) in the world (Figure 5 [the corrected form of Figure 7 in the original article]). Also, the corrected estimated incidence of type 1 diabetes was 0.020 (95% CI: 0.017 to 0.023, *P *< 0.001) in America (Figure 6 [the corrected form of Figure 6-A in the original article]). Moreover, in the abstract section of our paper, the estimate for the prevalence of type 1 diabetes should be 0.075% (95% CI: 0.051 to 0.110), instead of 9.5% (95% CI: 0.07 to 0.12). In summary, the overall results for the prevalence of type 1 diabetes in the world had a marginal change, and thus the conclusions drawn in our article are not changed.

## Disclosure

 As the team of authors, we take full responsibility for the errors and missing attributions, and appreciate the opportunity to prepare this corrigendum.

**Figure 1:**
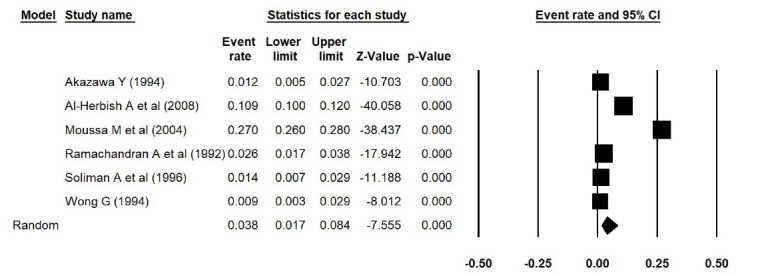
Prevalence of type 1 diabetes in Asia (the corrected form of Figure 2-B in the original article)

**Figure 2:**
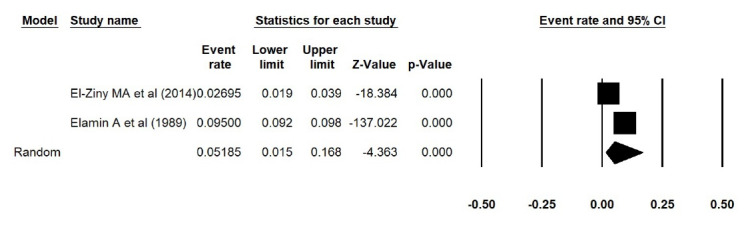
Prevalence of type 1 diabetes in Africa (the corrected form of Figure 3-B in the original article)

**Figure 3:**
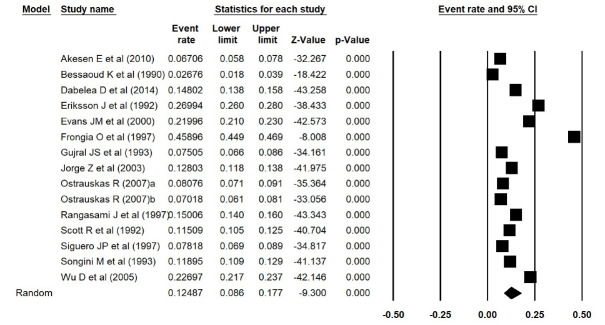
Prevalence of type 1 diabetes in Europe (the corrected form of Figure 5 in the original article)

**Figure 4:**
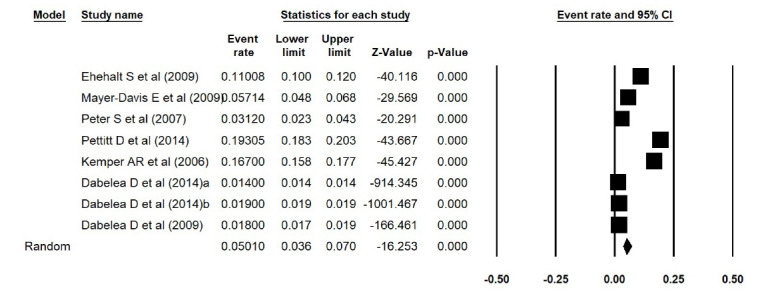
Prevalence of type 1 diabetes in America (the corrected form of Figure 6-B in the original article)

**Figure 5:**
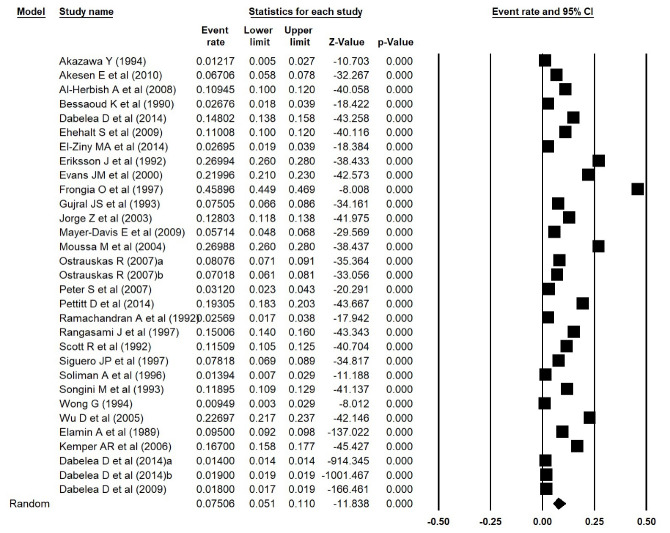
Prevalence of type 1 diabetes in the world (the corrected form of Figure 7 in the original article)

**Figure 6:**
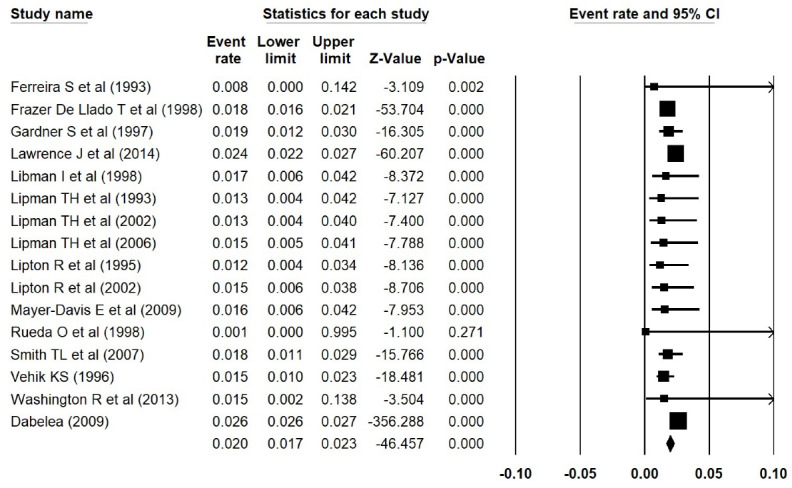
>Incidence of type 1 diabetes in America (the corrected form of Figure 6-A in the original article)
