# Supplementary material for: Prevalence and incidence of type 1 diabetes in the world: a systematic review and meta-analysis
Source: Health Promot Perspect. 2020 Mar 30;10(2):98–115. doi: 10.34172/hpp.2020.18 (PMC7146037; doi:10.34172/hpp.2020.18)
Supplement: Supplementary file 1 [file hpp-10-98-s001.pdf]

Supplementary file 1. Search Strategy of PubMed

Search	Query	Items found
#13	Search **((("Diabetes Mellitus, Type 1"[Mesh]) OR ((((((((((((((((((IDDM[Title/Abstract]) OR T1DM[Title/Abstract]) OR "Type 1 Diabetes"[Title/Abstract]) OR "Autoimmune Diabetes"[Title/Abstract]) OR "Juvenile Onset Diabetes"[Title/Abstract]) OR "Juvenile-Onset Diabetes"[Title/Abstract]) OR "Brittle Diabetes Mellitus"[Title/Abstract]) OR "brittle diabetes"[Title/Abstract]) OR "diabetes mellitus type 1"[Title/Abstract]) OR "diabetes mellitus type I"[Title/Abstract]) OR "diabetes type 1"[Title/Abstract]) OR "diabetes type I"[Title/Abstract]) OR "early onset diabetes mellitus"[Title/Abstract]) OR "insulin dependent diabetes"[Title/Abstract]) OR "juvenile diabetes"[Title/Abstract]) OR "juvenile diabetes mellitus"[Title/Abstract]) OR "type I diabetes"[Title/Abstract]) OR "type I diabetes mellitus"[Title/Abstract]) OR "Insulin Dependent Diabetes Mellitus"[Title/Abstract]) OR "Insulin-Dependent Diabetes Mellitus"[Title/Abstract]))) AND (("Incidence"[Mesh]) OR ((Incidence[Title/Abstract]) OR Incidences[Title/Abstract]))**	7621
#12	Search **((("Diabetes Mellitus, Type 1"[Mesh]) OR ((((((((((((((((((IDDM[Title/Abstract]) OR T1DM[Title/Abstract]) OR "Type 1 Diabetes"[Title/Abstract]) OR "Autoimmune Diabetes"[Title/Abstract]) OR "Juvenile Onset Diabetes"[Title/Abstract]) OR "Juvenile-Onset Diabetes"[Title/Abstract]) OR "Brittle Diabetes Mellitus"[Title/Abstract]) OR "brittle diabetes"[Title/Abstract]) OR "diabetes mellitus type 1"[Title/Abstract]) OR "diabetes mellitus type I"[Title/Abstract]) OR "diabetes type 1"[Title/Abstract]) OR "diabetes type I"[Title/Abstract]) OR "early onset diabetes mellitus"[Title/Abstract]) OR "insulin dependent diabetes"[Title/Abstract]) OR "juvenile diabetes"[Title/Abstract]) OR "juvenile diabetes mellitus"[Title/Abstract]) OR "type I diabetes"[Title/Abstract]) OR "type I diabetes mellitus"[Title/Abstract]) OR "Insulin Dependent Diabetes Mellitus"[Title/Abstract]) OR "Insulin-Dependent Diabetes Mellitus"[Title/Abstract]))) AND (("Prevalence"[Mesh]) OR ((Prevalence[Title/Abstract]) OR Prevalences[Title/Abstract]))**	7329
#11	Search **((("Diabetes Mellitus, Type 1"[Mesh]) OR ((((((((((((((((((IDDM[Title/Abstract]) OR T1DM[Title/Abstract]) OR "Type 1 Diabetes"[Title/Abstract]) OR "Autoimmune Diabetes"[Title/Abstract]) OR "Juvenile Onset Diabetes"[Title/Abstract]) OR "Juvenile-Onset Diabetes"[Title/Abstract]) OR "Brittle Diabetes Mellitus"[Title/Abstract]) OR "brittle diabetes"[Title/Abstract]) OR "diabetes mellitus type 1"[Title/Abstract]) OR "diabetes mellitus type**	13638

Search	Query	Items found
	**I"[Title/Abstract]) OR "diabetes type 1"[Title/Abstract]) OR "diabetes type I"[Title/Abstract]) OR "early onset diabetes mellitus"[Title/Abstract]) OR "insulin dependent diabetes"[Title/Abstract]) OR "juvenile diabetes"[Title/Abstract]) OR "juvenile diabetes mellitus"[Title/Abstract]) OR "type I diabetes"[Title/Abstract]) OR "type I diabetes mellitus"[Title/Abstract]) OR "Insulin Dependent Diabetes Mellitus"[Title/Abstract]) OR "Insulin-Dependent Diabetes Mellitus"[Title/Abstract]))) AND ((("Prevalence"[Mesh]) OR ((Prevalence[Title/Abstract]) OR Prevalences[Title/Abstract]))) OR (("Incidence"[Mesh]) OR ((Incidence[Title/Abstract]) OR Incidences[Title/Abstract])))**	
#10	Search **((("Prevalence"[Mesh]) OR ((Prevalence[Title/Abstract]) OR Prevalences[Title/Abstract]))) OR (("Incidence"[Mesh]) OR ((Incidence[Title/Abstract]) OR Incidences[Title/Abstract]))**	1415850
#9	Search **("Incidence"[Mesh]) OR ((Incidence[Title/Abstract]) OR Incidences[Title/Abstract])**	827503
#8	Search **(Incidence[Title/Abstract]) OR Incidences[Title/Abstract]**	727288
#7	Search **"Incidence"[Mesh]**	248745
#6	Search **("Prevalence"[Mesh]) OR ((Prevalence[Title/Abstract]) OR Prevalences[Title/Abstract])**	671215
#5	Search **(Prevalence[Title/Abstract]) OR Prevalences[Title/Abstract]**	590477
#4	Search **"Prevalence"[Mesh]**	275324
#3	Search **("Diabetes Mellitus, Type 1"[Mesh]) OR ((((((((((((((((((IDDM[Title/Abstract]) OR T1DM[Title/Abstract]) OR "Type 1 Diabetes"[Title/Abstract]) OR "Autoimmune Diabetes"[Title/Abstract]) OR "Juvenile Onset Diabetes"[Title/Abstract]) OR "Juvenile-Onset Diabetes"[Title/Abstract]) OR "Brittle Diabetes Mellitus"[Title/Abstract]) OR "brittle diabetes"[Title/Abstract]) OR "diabetes mellitus type 1"[Title/Abstract]) OR "diabetes mellitus type I"[Title/Abstract]) OR "diabetes type 1"[Title/Abstract]) OR "diabetes type I"[Title/Abstract]) OR "early onset diabetes mellitus"[Title/Abstract]) OR "insulin dependent diabetes"[Title/Abstract]) OR "juvenile diabetes"[Title/Abstract]) OR "juvenile diabetes mellitus"[Title/Abstract]) OR "type I diabetes"[Title/Abstract]) OR "type I diabetes mellitus"[Title/Abstract]) OR "Insulin Dependent Diabetes Mellitus"[Title/Abstract]) OR "Insulin-Dependent Diabetes Mellitus"[Title/Abstract])**	99341
#2	Search **((((((((((((((((((IDDM[Title/Abstract]) OR T1DM[Title/Abstract]) OR "Type 1 Diabetes"[Title/Abstract]) OR "Autoimmune Diabetes"[Title/Abstract]) OR "Juvenile Onset Diabetes"[Title/Abstract]) OR "Juvenile-Onset Diabetes"[Title/Abstract]) OR "Brittle Diabetes**	68175

Search	Query	Items found
	**Mellitus"[Title/Abstract]) OR "brittle diabetes"[Title/Abstract]) OR "diabetes mellitus type 1"[Title/Abstract]) OR "diabetes mellitus type I"[Title/Abstract]) OR "diabetes type 1"[Title/Abstract]) OR "diabetes type I"[Title/Abstract]) OR "early onset diabetes mellitus"[Title/Abstract]) OR "insulin dependent diabetes"[Title/Abstract]) OR "juvenile diabetes"[Title/Abstract]) OR "juvenile diabetes mellitus"[Title/Abstract]) OR "type I diabetes"[Title/Abstract]) OR "type I diabetes mellitus"[Title/Abstract]) OR "Insulin Dependent Diabetes Mellitus"[Title/Abstract]) OR "Insulin-Dependent Diabetes Mellitus"[Title/Abstract]**	
#1	Search **"Diabetes Mellitus, Type 1"[Mesh]**	73750

Search Strategy of Embase

#13
#3 AND #9
11,162
#12
#3 AND #6
12,085
#11
#3 AND #10
21,010
#10
#6 OR #9
2,051,248
#9
#7 OR #8
1,174,867
#8
incidence*:ab,ti
1,038,681
#7
'incidence'/exp
444,375
#6
#4 OR #5
1,004,060
#5
prevalence*:ab,ti
831,765
#4
'prevalence'/exp

695,313
#3
#1 OR #2
140,799
#2
iddm:ab,ti OR t1dm:ab,ti OR 'type 1 diabetes':ab,ti OR 'autoimmune diabetes':ab,ti OR 'juvenile onset diabetes':ab,ti OR 'juvenile-onset diabetes':ab,ti OR 'brittle diabetes mellitus':ab,ti OR 'brittle diabetes':ab,ti OR 'diabetes mellitus type 1':ab,ti OR 'diabetes mellitus type i':ab,ti OR 'diabetes type 1':ab,ti OR 'diabetes type i':ab,ti OR 'early onset diabetes mellitus':ab,ti OR 'insulin dependent diabetes':ab,ti OR 'juvenile diabetes':ab,ti OR 'juvenile diabetes mellitus':ab,ti OR 'type i diabetes':ab,ti OR 'type i diabetes mellitus':ab,ti OR 'insulin dependent diabetes mellitus':ab,ti OR 'insulin-dependent diabetes mellitus':ab,ti
96,781
#1
'insulin dependent diabetes mellitus'/exp
113,761
